# Alpha-2-Macroglobulin-Enriched Plasma Modulates Inflammatory and Proteolytic Pathways in Experimental Equine Musculoskeletal Models

**DOI:** 10.3390/ani16162595

**Published:** 2026-08-19

**Authors:** Enrico Gugliandolo, Gianluca Antonio Franco, Vito Biondi, Maria De Luca, Yanne Van Reusel, Francesco Tosto, Francesca Inferrera, Giuseppe Catone, Jan H. Spaas

**Affiliations:** 1 Department of Veterinary Sciences, University of Messina, 98168 Messina, Italy; giafranco@studenti.unime.it (G.A.F.); vito.biondi@unime.it (V.B.); mari.deluca.96@gmail.com (M.D.L.); gcatone@unime.it (G.C.); 2 Department of Research and Development, INTIBIO, 3960 Bree, Belgiumjan@intibio.com (J.H.S.); 3 Department of Morphology, Imaging, Orthopedics, Rehabilitation and Nutrition, Faculty of Veterinary Medicine, Ghent University, 9820 Merelbeke, Belgium; 4 Independent Researcher, 98166 Messina, Italy; tostodotf@libero.it; 5 Department CHIBIOFARAM, University of Messina, 98166 Messina, Italy; francesca.inferrera@studenti.unime.it

**Keywords:** alpha-2-macroglobulin, orthobiologics, equine osteoarthritis, synovitis, chondrocytes, synoviocytes, extracellular matrix, tendon explants, matrix metalloproteinase-13, regenerative medicine

## Abstract

Joint inflammation and extracellular matrix degradation are major contributors to the development of equine osteoarthritis and reduced athletic performance. Alpha-2-macroglobulin is a naturally occurring broad-spectrum inhibitor of proteases and inflammatory mediators that has emerged as a promising orthobiologic therapy. This study investigated the anti-inflammatory and matrix-protective effects of alpha-2-macroglobulin-enriched plasma in complementary in vitro and ex vivo equine models. Primary equine chondrocytes and synoviocytes isolated from healthy horses were stimulated with interleukin-1β or lipopolysaccharide and treated with alpha-2-macroglobulin-enriched plasma or matched platelet-poor plasma. In parallel, an equine tendon explant model challenged with collagenase was used to evaluate extracellular matrix preservation. Alpha-2-macroglobulin-enriched plasma significantly reduced the production of tumor necrosis factor-α, interleukin-6, and matrix metalloproteinase-13, downregulated interleukin-1β gene expression, and partially restored collagen type II expression in inflamed joint cells compared with platelet-poor plasma. In tendon explants, treatment preserved tissue architecture, reduced collagen degradation, and improved histological indices of matrix integrity under proteolytic challenge. These findings demonstrate that alpha-2-macroglobulin-enriched plasma exerts anti-inflammatory, anti-catabolic, and matrix-protective effects in experimental equine tissues. The results provide mechanistic support for the development of alpha-2-macroglobulin-based orthobiologic therapies as a complementary strategy for the management of synovitis and early osteoarthritis in horses.

## Simple Summary

Joint inflammation is one of the leading causes of pain, reduced athletic performance, and early retirement in sport horses. Current treatments often relieve clinical signs but may not adequately protect joint tissues from ongoing damage. Alpha-2-macroglobulin is a naturally occurring blood protein that can bind and neutralize enzymes and inflammatory molecules involved in cartilage destruction. In this study, we evaluated a blood-derived preparation enriched in alpha-2-macroglobulin using cells and tissues obtained from healthy horses. The treatment reduced the production of inflammatory molecules, decreased the release of enzymes responsible for cartilage breakdown, and helped preserve the structure of collagen-rich tissues exposed to damaging conditions. These findings suggest that alpha-2-macroglobulin-enriched plasma may provide a biological approach to support joint health by reducing inflammation while protecting the extracellular matrix. Although further studies in horses with naturally occurring joint disease are needed, these results provide experimental evidence supporting the future development of this therapy as a complementary treatment for equine joint disorders, with the potential to improve animal welfare, prolong athletic performance, and reduce the progression of degenerative joint disease.

## 1. Introduction

Alpha-2-macroglobulin (A2MG) is a large plasma glycoprotein that has been known since the 1950s and is widely recognized for its exceptional ability to neutralize proteolytic enzymes [1]. Structurally, A2MG is a tetramer composed of four subunits of approximately 180 kDa, each containing a flexible “bait region”. When this region is cleaved by proteases—such as matrix metalloproteinases (MMPs), plasmin, elastase, or trypsin—the molecule undergoes a conformational rearrangement that physically entraps the enzyme. The resulting A2MG–protease complex is then rapidly removed from the extracellular space through interaction with the low-density lipoprotein receptor-related protein 1 (LRP1), thereby preventing uncontrolled proteolytic activity [2]. In addition to its antiprotease function, A2MG plays an important modulatory role in inflammation. It can bind pro-inflammatory cytokines, including interleukin-1β (IL-1β) and tumour necrosis factor-α (TNF-α), reducing their bioavailability and downstream signalling [3]. These cytokine-bound complexes are likewise cleared via LRP1-mediated endocytosis [4], providing a physiological mechanism for limiting the persistence of catabolic mediators and contributing to extracellular-matrix homeostasis [5]. Although the biology of A2MG has been extensively investigated in humans and laboratory species, its functional and translational relevance in the equine joint—particularly within the stifle—remains relatively underexplored. In horses, A2MG is mainly synthesized by the liver and circulates in plasma at concentrations of approximately 0.2–0.4 mg·mL^−1^, while markedly lower levels are found in synovial fluid [6]. Within the joint environment, A2MG is thought to act as a local protective factor, buffering excess proteases and inflammatory mediators released during synovitis or mechanical overload, and thereby supporting the structural integrity of cartilage and synovial membrane [7,8]. Emerging evidence also suggests that A2MG, together with other zinc-binding proteins such as metallothioneins, may influence immune regulation and oxidative-stress responses, linking protease control to broader mechanisms of tissue homeostasis [9,10,11]. With ageing or chronic inflammatory disease, however, these protective pathways may become dysregulated, potentially shifting from a homeostatic role toward one that contributes to progressive tissue degeneration [12]. The combination of broad antiproteolytic and anti-inflammatory actions has therefore positioned A2MG as an attractive biologic candidate for the management of equine joint disease [13]. Synovitis of the equine stifle exemplifies the close interplay between inflammation and matrix degradation: cytokines released by the synovial lining stimulate protease activity, leading to cartilage and collagen breakdown, while degradation products further amplify inflammation in a self-perpetuating cycle. Conventional intra-articular therapies, such as corticosteroids and hyaluronic acid, are effective at reducing pain and effusion but rarely disrupt this inflammatory–catabolic loop or provide sustained protection of the extracellular matrix [14,15]. Within this context, and in line with our broader research strategy aimed at supporting endogenous pro-homeostatic mechanisms, we hypothesized that allogeneic α_2_-macroglobulin-enriched plasma (A2MG-EP) could attenuate inflammatory signalling and limit matrix catabolism in equine joint tissues. To test this hypothesis, we evaluated the effects of A2MG-EP in complementary experimental models, including primary equine chondrocytes and synoviocytes stimulated with IL-1β or lipopolysaccharide (LPS). Because the primary biological function of α_2_-macroglobulin is the sequestration and inactivation of a wide range of proteases—including collagenases and matrix metalloproteinases—we also incorporated an ex vivo tendon explant model as a mechanistic complement to the cellular assays. Tendon extracellular matrix is particularly rich in fibrillar collagen and highly susceptible to protease-driven degradation, making it a sensitive and reductionist system in which to assess anti-proteolytic activity at the tissue level. This model allows direct structural evaluation of collagen preservation and provides functional confirmation of α_2_-macroglobulin’s protease-neutralizing capacity, independent of joint-specific cellular responses. Beyond its mechanistic value, tendon tissue also carries translational relevance, as protease-mediated matrix degradation represents a shared pathological feature of both intra-articular soft-tissue and tendon injuries in athletic horses. Although tendon pathology was not the primary focus of the present study, these considerations underscore the broader potential of α_2_-macroglobulin-based strategies in equine musculoskeletal disease. Overall, this work aims to provide convergent mechanistic and translational evidence supporting A2MG as a biologic modulator of inflammation and extracellular-matrix integrity in the equine joint, thereby informing the design of future controlled clinical studies. We therefore aimed to determine whether characterised A2MG-EP could modulate inflammatory responses in equine joint-derived cells and attenuate collagenase-mediated extracellular-matrix degradation in tendon explants. The study was designed as a preliminary mechanistic evaluation of the complete plasma-derived preparation and not as an assessment of purified A2MG.

## 2. Materials and Methods

### 2.1. Tissue Processing

Immediately post mortem at an EU-licensed abattoir, femoropatellar and femorotibial joints from six adult Warmblood horses aged 6–8 years (slaughtered for food purposes; procedures unrelated to this study) were aseptically opened on the processing line. Articular cartilage surfaces were macroscopically inspected and confirmed to be free from osteoarthritic changes including erosion, discolouration and fibrillation. Similarly, synovial membranes showed no gross evidence of hyperaemia, villous hypertrophy or fibrosis. Using a sterile 4 mm biopsy punch (Integra, Saint Priest, France), cartilage explants were harvested from the medial femoral condyle and 10 synovial membrane explants were collected from the dorsal and plantar recesses of each joint. Explants were immediately immersed in ice-cold Dulbecco’s Modified Eagle Medium (DMEM) supplemented with 10% fetal bovine serum and 1% penicillin–streptomycin, maintained at 4 °C and transported to the laboratory within 90 min, as previously described [16]. Superficial digital flexor tendons were exposed in situ within 60 min after exsanguination. Full-thickness longitudinal cores (4 mm diameter, 5 mm length) were obtained from the mid-metacarpal region, trimmed with a sterile scalpel, gently blotted and individually placed in sterile tubes containing ice-cold Hank’s Balanced Salt Solution supplemented with 2% (*v*/*v*) antibiotic–antimycotic solution (Thermo Fisher Scientific, Waltham, MA, USA). Only the punched cores, rather than entire tendon segments, were transported. All tendon samples reached the laboratory within 2 h of collection, in accordance with established protocols [17]. Upon arrival, cartilage explants were trimmed with a No. 10 scalpel blade to remove the calcified zone, whereas synovial membrane explants were dissected under a stereomicroscope to remove residual fibrous capsule tissue. Tendon cores required no additional processing. For primary cell isolation, cartilage explants were minced and digested with collagenase type II (0.75 mg/mL in DMEM) for 12–16 h at 37 °C under gentle agitation, as previously described for equine articular cartilage [18,19]. Synovial membrane explants were minced and digested with collagenase type II (0.75 mg/mL in DMEM) for 4 h at 37 °C under gentle agitation according to established protocols for equine synovial tissue [18,19]. Cell suspensions were filtered through a 70 μm cell strainer, centrifuged and resuspended in complete DMEM supplemented with 10% fetal bovine serum and 1% penicillin–streptomycin. Primary equine chondrocytes and synoviocytes were cultured at 37 °C in a humidified atmosphere containing 5% CO_2_ and expanded to passages 2–3 before experimental use to minimise phenotypic drift.

### 2.2. Experimental Design and Replicates

For in vitro experiments, primary equine chondrocytes and synoviocytes were isolated from six independent horses. The tissue-donor horse was considered the biological replicate and the experimental unit for statistical analysis. Technical replicates (duplicate wells per condition) were averaged before statistical analysis and were not treated as independent observations. For the ex vivo tendon assay, superficial digital flexor tendon explants were obtained from the same six tissue-donor horses. Multiple tendon cores were processed from each horse; however, measurements were averaged to generate one value per horse per condition, such that the tissue-donor horse represented the experimental unit. Therefore, the final sample size for statistical analysis was *n* = 6 independent biological replicates per condition. α_2_-Macroglobulin-enriched plasma (A2MG-EP) using a standardized commercial kit (A2MG, INTIBIO, Bree, Belgium) according to the manufacturer’s instructions. Equal volumes from the five donor-derived preparations were combined to generate a single pooled A2MG-EP preparation to minimise donor-specific variability and approximate a standardized allogeneic product [20]. Whole blood was collected from five donor horses that were different from the six horses used for primary cell isolation and tendon explant collection. The five plasma donors were male, 9–12 years old and were considered clinically healthy at the time of blood collection on the basis of a physical examination. This exploratory proof-of-concept study included six independent tissue-donor horses, consistent with previous investigations using primary equine cells and ex vivo musculoskeletal tissues [16,17,18]. The sample size was considered appropriate for detecting large, biologically consistent effects under controlled in vitro and ex vivo conditions, but the study was not designed to detect small effects or establish clinical efficacy. The tissue-donor horse was considered the biological replicate and experimental unit. Measurements from multiple wells, explants, histological sections, microscopic fields, or technical assay replicates originating from the same horse were averaged before statistical analysis and were not treated as independent observations. The final biological sample size was therefore *n* = 6 independent horses.

### 2.3. Preparation of α_2_-Macroglobulin-Enriched Plasma (A2MG-EP)

Whole blood samples were processed using a commercially available A2MG preparation kit (INTIBIO, Bree, Belgium), which, according to the manufacturer’s specifications, yields a plasma fraction enriched in α_2_-macroglobulin with minimal cellular and platelet contamination [20]. The performance and compositional effects of this preparation platform have been extensively investigated in our previous study by Van Reusel et al. [20]. In that study, preparations independently generated from six healthy Warmblood horses were characterised using haematological, biochemical, and immunoassay-based analyses. The processing procedure resulted in marked depletion of blood cells and platelets, enrichment of A2MG, and a biochemical profile distinct from baseline blood and PRP-derived preparations [20]. The same commercial preparation platform was used in the present study. Briefly, two 50 mL syringes were each preloaded with 5 mL anticoagulant citrate dextrose solution A (ACD-A; final concentration 10% *v*/*v*). A total of 70 mL blood (35 mL per syringe) was collected from the jugular vein of each donor horse and gently inverted several times to ensure homogeneous mixing with the anticoagulant. For the first centrifugation step, anticoagulated blood was transferred into the designated separation tubes and centrifuged at 1400× g for 10 min at 20 °C. Following centrifugation, the platelet-poor plasma (PPP) fraction was aseptically collected into a sterile 50 mL Luer-lock syringe. For α_2_-macroglobulin enrichment, the plasma fraction was transferred into adsorption tubes and centrifuged again at 2160× g for 10 min. The resulting supernatant, corresponding to α_2_-macroglobulin-enriched plasma (A2MG-EP), was collected aseptically using the accessories supplied by the manufacturer. No sample underwent more than one freeze–thaw cycle. PPP was prepared using a double-spin protocol (1400× g for 10 min followed by 1400× g for 10 min; low brake), collecting the upper two-thirds of the plasma fraction. PPP was volume- and anticoagulant-matched to A2MG-EP. Following pooling and before experimental use, the final A2MG-EP and PPP preparations underwent targeted specific compositional characterisation. A2MG concentration was quantified using a commercially available equine-specific sandwich ELISA kit (MyBioSource, San Diego, CA, USA; catalogue no. MBS014509) according to the manufacturer’s instructions. Samples were analysed in technical duplicate, optical density was measured at 450 nm, and concentrations were calculated from a four-parameter logistic standard curve after application of the appropriate sample-dilution factor. A2MG concentrations were 1.58 mg/mL in A2MG-EP and 0.47 mg/mL in PPP. Total protein concentration, determined using a bicinchoninic acid assay, was 6.95 g/dL in A2MG-EP and 5.38 g/dL in PPP. Residual platelet counts, determined using an automated haematology analyser, were 2.5 × 10^9^/L and 5.5 × 10^9^/L, respectively. Because the five plasma donors were different from the six tissue donors, the pooled preparations were allogeneic with respect to the cells and tissues used in the experimental models. No preparation underwent more than one freeze–thaw cycle.

### 2.4. Cell Viability Assay

Primary equine chondrocytes and synoviocytes (passages 2–3) were seeded in 24-well plates at a density of 1 × 10^5^ cells/well and cultured in antibiotic-free Dulbecco’s Modified Eagle Medium supplemented with 10% fetal bovine serum. Cells were allowed to adhere and proliferate for 48 h. Cells were subsequently exposed to increasing concentrations (0%, 5%, 10% and 20% *v*/*v*) of A2MG-EP or matched PPP, as described above. Treatments were performed in serum-free medium and maintained for 24 h at 37 °C in a humidified atmosphere containing 5% CO_2_. Cell viability was evaluated using the MTT assay. Briefly, MTT solution (0.5 mg/mL; Sigma-Aldrich, St. Louis, MO, USA) was added to each well and incubated for 2 h at 37 °C. The medium was then removed and the resulting formazan crystals were dissolved in 300 μL dimethyl sulfoxide with gentle agitation for 15 min. Absorbance was measured at 570 nm using a microplate reader (Multiskan, Thermo Fisher Scientific, Waltham, MA, USA). Cell viability was expressed as the percentage of metabolic activity relative to untreated control cells maintained in serum-free medium.

### 2.5. Inflammatory Challenge and Treatment in Vitro

Primary equine articular chondrocytes and synoviocytes (passages 2–3) were seeded at a density of 1 × 10^5^ cells/well in 24-well plates and cultured for 48 h in complete Dulbecco’s Modified Eagle Medium (DMEM; Sigma-Aldrich, St. Louis, MO, USA) supplemented with 10% fetal bovine serum (FBS) and 1% penicillin–streptomycin. After 48 h, cells were washed twice with sterile phosphate-buffered saline (PBS). Cells were then incubated for 24 h in serum-free DMEM containing IL-1β (2 ng/mL) or LPS (10 ng/mL), either alone or in combination with A2MG-EP or matched PPP at final concentrations of 10% or 20% (*v*/*v*). Following 24 h of exposure, culture supernatants were collected and clarified by centrifugation (10,000× *g*, 10 min, 4 °C) before storage at −80 °C for cytokine and protease analyses. Cell monolayers were washed twice with PBS and stored at −80 °C for subsequent RNA extraction and gene expression analysis.

### 2.6. Enzyme-Linked Immunosorbent Assays

Samples were analysed in duplicate using equine-specific sandwich enzyme-linked immunosorbent assay kits. The following analytes were quantified: tumour necrosis factor-α (Cloud-Clone Corp., SEA133Eq. Houston, TX, USA), interleukin-6 (Cloud-Clone Corp., SEA079Eq. Houston, TX, USA) and Matrix Metalloproteinase 13 (MMP13 Cloud-Clone Corp., SEA099Eq. Houston, TX, USA). All assays were performed according to the manufacturers’ instructions. Absorbance was measured at 450 nm using a Multiskan microplate reader (Thermo Fisher Scientific, Waltham, MA, USA). Concentrations were calculated from standard curves generated using four-parameter logistic regression in GraphPad Prism version 10 (GraphPad Software, San Diego, CA, USA). All ELISA measurements were performed in technical duplicate. The mean of the two technical measurements was calculated for each biological replicate and experimental condition before statistical analysis. Technical replicates were not considered independent observations.

### 2.7. Ex Vivo Tendon Degradation Assay

The tendon explant assay was designed as a protease-dominant, collagen-rich ex vivo model to evaluate the anti-proteolytic activity of α_2_-macroglobulin-enriched plasma at the tissue level. Upon arrival at the laboratory, full-thickness longitudinal tendon cores (4 mm diameter, 5 mm length) were inspected and allocated to treatment groups using a computer-generated randomization sequence. Explants were incubated in 24-well plates containing 1 mL serum-free DMEM supplemented with collagenase type II (0.5 mg/mL; approximately 500 U/mL) to induce acute extracellular matrix degradation. α_2_-Macroglobulin-enriched plasma was added simultaneously at a final concentration of 20% (*v*/*v*). Control explants were incubated with collagenase in the presence of an equivalent volume of matched platelet-poor plasma (20% *v*/*v*). After 24 h at 37 °C, explants were washed twice with PBS and processed for biochemical and histological analyses. Corresponding culture supernatants were collected for biochemical evaluation. Superficial digital flexor tendon explants were fixed in 4% paraformaldehyde, dehydrated and embedded in paraffin. Transverse sections (5 μm) were stained with haematoxylin and eosin. Images were acquired at 10× and 20× magnification using identical acquisition settings for all groups. For each explant (*n* = 6), three non-adjacent sections were analysed. From each section, 10 non-overlapping microscopic fields were selected using systematic random sampling. Each field was subdivided into three regions of interest (ROIs). Image analysis was performed using Fiji (ImageJ v1.54t; National Institutes of Health, Bethesda, MD, USA). The eosin channel was extracted by colour deconvolution according to Ruifrok and Johnston [21].

Fibre density (FD, %) was calculated as:

FD = (1 − Nbackground/NROI) × 100(1)

The interfascicular space area (%) was quantified as the proportion of background pixels. ROI measurements were averaged to obtain one value per field, and field values were averaged to generate a single value per explant. Results were normalized to the mean value of untreated tendon explants, which was set to 100% [22]. Collagen content was quantified by hydroxyproline determination using a commercial colorimetric assay kit (Sigma-Aldrich, MAK008) according to the manufacturer’s instructions [23].

### 2.8. Quantitative Real-Time Polymerase Chain Reaction Analysis

Total RNA was extracted from equine chondrocytes and synoviocytes using the RNeasy Mini Kit (Qiagen, Hilden, Germany) according to the manufacturer’s instructions. RNA concentration and purity were assessed using a NanoDrop spectrophotometer (Thermo Fisher Scientific, Waltham, MA, USA). Reverse transcription was performed using 1 μg total RNA and the RevertAid First Strand cDNA Synthesis Kit (Thermo Fisher Scientific) with random hexamer primers according to the manufacturer’s instructions. Quantitative real-time polymerase chain reaction was performed using SsoAdvanced Universal SYBR Green Supermix (Bio-Rad Laboratories, Hercules, CA, USA) in 20 μL reaction volumes (Table 1) on a CFX96 Touch Real-Time PCR Detection System (Bio-Rad Laboratories). Melting curve analysis (65–95 °C; 0.5 °C increments) was performed following amplification to verify product specificity. All reactions were performed in technical triplicate, and no-template controls were included to monitor contamination. Relative gene expression was calculated using the 2^−ΔΔCt^ method. Gene expression values were normalized to glyceraldehyde-3-phosphate dehydrogenase (GAPDH) and expressed as fold change relative to untreated controls [24]. Quantitative PCR reactions were performed in technical triplicate. Technical Ct values were averaged before calculation of ΔCt values for each biological replicate. Statistical analyses were performed on ΔCt values, with the individual tissue-donor horse considered the experimental unit.

### 2.9. Statistical Analysis

All statistical analyses were performed using GraphPad Prism version 10 (GraphPad Software, San Diego, CA, USA). Data are presented as mean ± standard error of the mean (SEM). The tissue-donor horse was considered the biological replicate and experimental unit for all analyses. For in vitro experiments, measurements obtained from primary cell cultures derived from the same horse were averaged before analysis. For ex vivo experiments, measurements obtained from multiple tendon explants originating from the same horse were averaged to generate a single value per horse and treatment condition. Technical replicates were averaged before statistical analysis and were not treated as independent observations. Because each donor horse contributed samples to multiple experimental conditions, observations were analysed as matched rather than independent data. For each outcome, donor-level values were analysed using a repeated-measures mixed-effects model fitted by restricted maximum likelihood (REML) in GraphPad Prism version 10, with experimental condition specified as the fixed effect and donor horse included as the matching/random effect. Geisser–Greenhouse correction was applied to the omnibus test. Tukey-adjusted multiple comparisons were subsequently performed. Technical replicates and measurements from multiple explants originating from the same donor horse under the same experimental condition were averaged to generate one donor-level value before statistical analysis. For quantitative real-time polymerase chain reaction analyses, statistical testing was performed on ΔCt values, whereas relative expression is presented as 2^−ΔΔCt^. Adjusted *p* values < 0.05 were considered statistically significant.

## 3. Results

### 3.1. A2MG-EP Does Not Affect Cell Viability In Vitro

**Figure 1:**
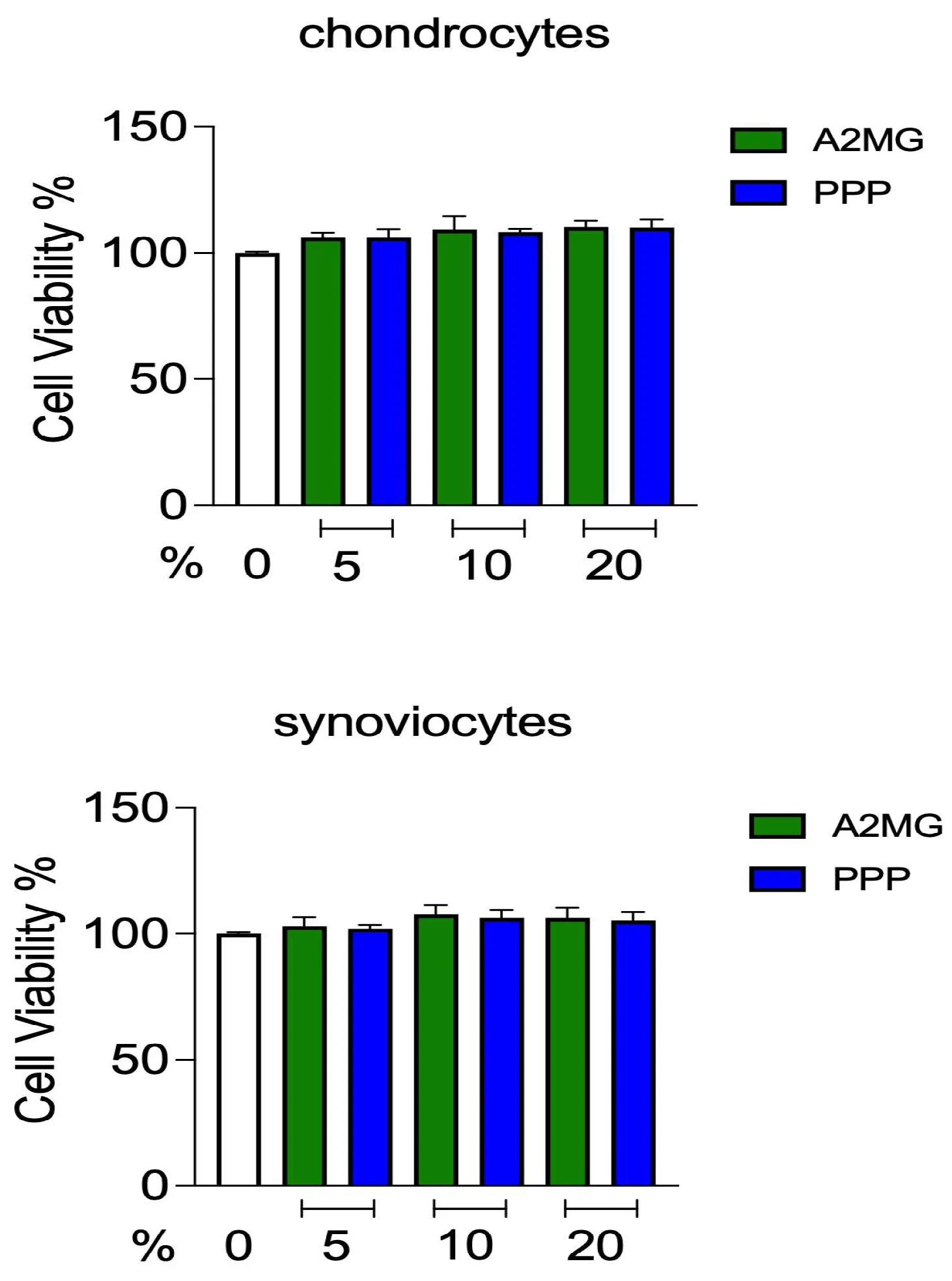
Effect of α_2_-macroglobulin-enriched plasma (A2MG-EP) on the viability of primary equine chondrocytes and synoviocytes. Cells isolated from six independent donor horses (*n* = 6) were treated for 24 h with 0% (serum-free Dulbecco’s Modified Eagle Medium), 5%, 10% or 20% (*v*/*v*) A2MG-EP or matched platelet-poor plasma (PPP). Cell viability was assessed using the MTT assay and expressed as a percentage of untreated control cells. Data are presented as mean ± SEM. Statistical analysis was performed using a REML mixed-effects model with donor horse as the matching factor, followed by Geisser–Greenhouse correction and Tukey-adjusted multiple comparisons. (*p* > 0.05).

Exposure of primary equine articular chondrocytes and synoviocytes to α_2_-macroglobulin-enriched plasma (A2MG-EP) at 5–20% (*v*/*v*) for 24 h did not decrease MTT metabolic activity compared with untreated controls (Figure 1). Across all concentrations, viability values remained close to baseline and well above the ISO 10993-5 non-cytotoxicity threshold (70%) [25], indicating that the formulation is not cytotoxic under the conditions tested. Matched platelet-poor plasma (PPP) tested at identical dilutions showed a comparable profile, with no significant differences relative to untreated controls (REML mixed-effects model with experimental condition as a fixed effect and donor horse as a random effect, followed by Tukey’s multiple-comparisons test with the Geisser–Greenhouse correction; *p* > 0.05). These data support the use of 10–20% (*v*/*v*) A2MG-EP in subsequent assays without evidence of confounding cytotoxicity.

### 3.2. Effects in Equine Chondrocytes

**Figure 2:**
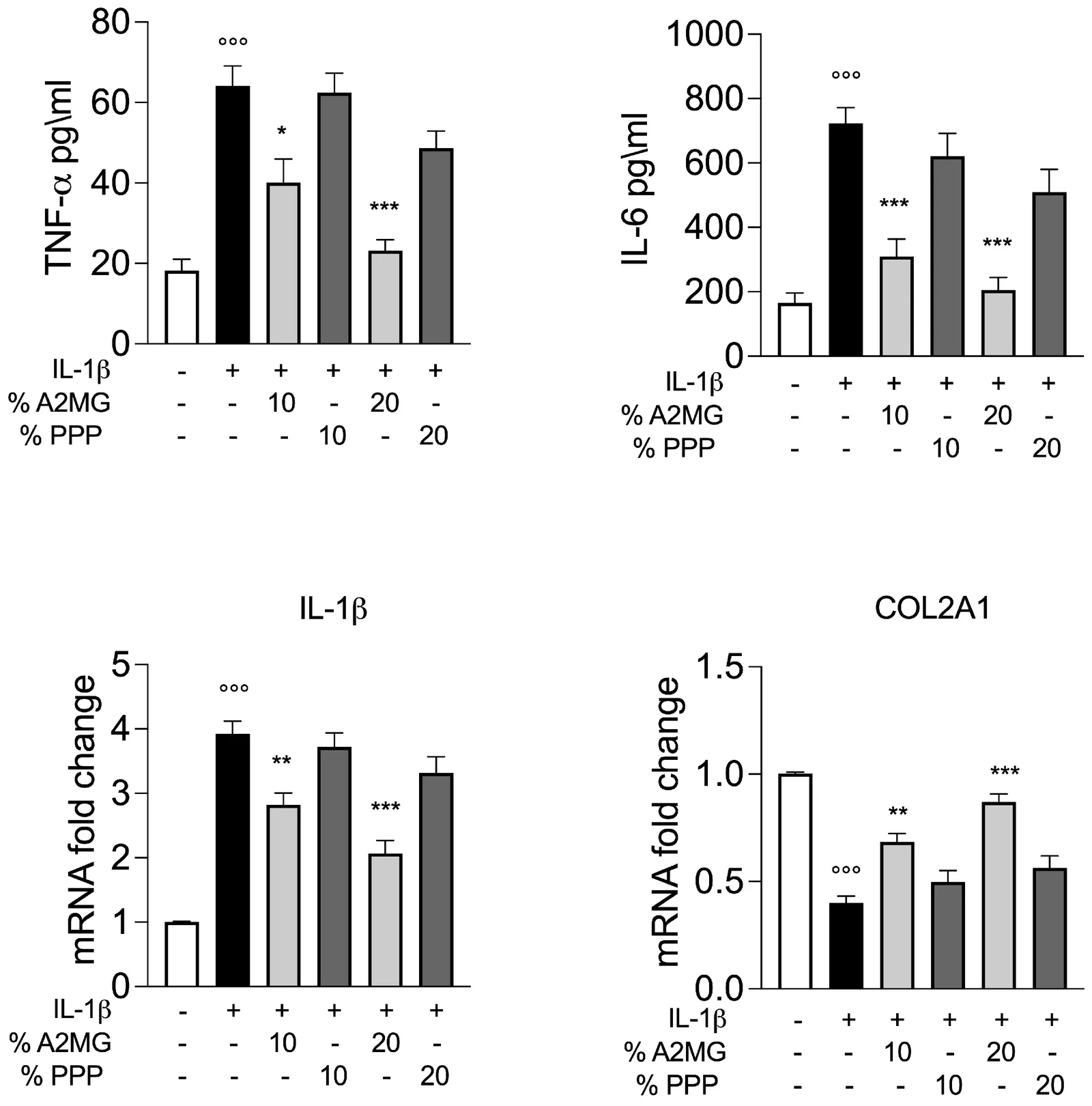
Effects of α_2_-macroglobulin-enriched plasma (A2MG-EP) on IL-1β-induced inflammatory responses in primary equine chondrocytes. Chondrocytes isolated from six independent donor horses (*n* = 6) were stimulated with IL-1β (2 ng/mL) for 24 h in the presence or absence of A2MG-EP or matched platelet-poor plasma (PPP) at 10% or 20% (*v*/*v*). TNF-α and IL-6 concentrations were measured in culture supernatants (upper panels). IL-1β and COL2A1 mRNA expression were determined by quantitative real-time polymerase chain reaction and expressed as fold change relative to untreated controls (lower panels). Data are presented as mean ± SEM. Statistical analysis was performed using a REML mixed-effects model with donor horse as the matching factor, followed by Geisser–Greenhouse correction and Tukey-adjusted multiple comparisons.°°° *p* < 0.001 vs. CTR; * *p* < 0.05 vs. IL-1β; ** *p* < 0.01 vs. IL-1β; *** *p* < 0.001 vs. IL-1β.

**Figure 3:**
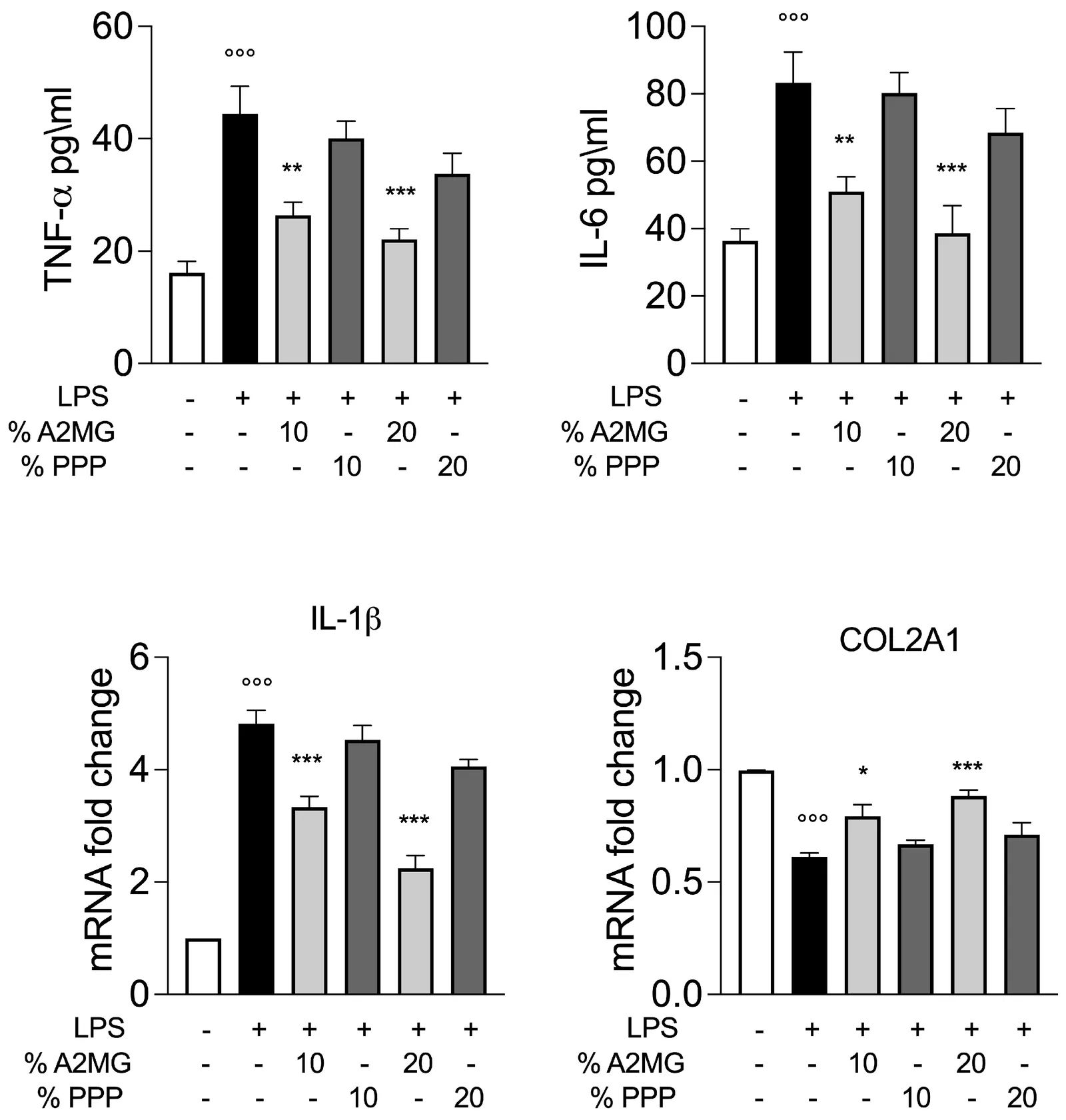
Effects of α_2_-macroglobulin-enriched plasma (A2MG-EP) on lipopolysaccharide-induced inflammatory responses in primary equine chondrocytes. Chondrocytes isolated from six independent donor horses (*n* = 6) were stimulated with lipopolysaccharide (10 ng/mL) for 24 h in the presence or absence of A2MG-EP or matched platelet-poor plasma (PPP) at 10% or 20% (*v*/*v*). TNF-α and IL-6 concentrations were measured in culture supernatants (upper panels). IL-1β and COL2A1 mRNA expression were determined by quantitative real-time polymerase chain reaction and expressed as fold change relative to untreated controls (lower panels). Data are presented as mean ± SEM. Statistical analysis was performed using a REML mixed-effects model with donor horse as the matching factor, followed by Geisser–Greenhouse correction and Tukey-adjusted multiple comparisons.°°° *p* < 0.001 vs. CTR; * *p* < 0.05 vs. LPS; ** *p* < 0.01 vs. LPS; *** *p* < 0.001 vs. LPS.

Both IL-1β (2 ng/mL, 24 h) and LPS (10 ng/mL, 24 h) induced a robust inflammatory response in primary equine chondrocytes. Stimulation with either agent significantly increased TNF-α (*p* = 0.0001 vs. CTR) and IL-6 (*p* < 0.0001 vs. CTR) secretion, accompanied by a parallel up-regulation of IL-1β (*p* < 0.0001 vs. CTR) mRNA expression (Figure 2 and Figure 3). In parallel, expression of COL2A1, a marker of the chondrogenic phenotype, was markedly reduced following inflammatory stimulation (*p* < 0.0001 vs. CTR). Co-treatment with α_2_-macroglobulin-enriched plasma (A2MG-EP) attenuated these inflammatory responses in a concentration-dependent manner. A2MG-EP 10% significantly reduced the secretion of TNF-α (*p* = 0.0251 vs. IL-1β; *p* = 0.0038 vs. LPS) and IL-6 (*p* = 0.0006 vs. IL-1β; *p* = 0.0078 vs. LPS), as well as the induction of IL-1β transcripts (*p* = 0.0046 vs. IL-1β; *p* = 0.0005 vs. LPS), compared with stimulated controls. The higher concentration of A2MG-EP (20% *v*/*v*) produced the strongest effect on TNF-α (*p* = 0.0009 vs. IL-1β; *p* = 0.0001 vs. LPS), IL-6 (*p* = 0.0002 vs. IL-1β; *p* = 0.0003 vs. LPS), IL-1β transcripts (*p* = 0.0005 vs. IL-1β; *p* < 0.0001 vs. LPS), and partially restored COL2A1 expression toward basal levels (*p* < 0.0001 vs. IL-1β; *p* = 0.0002 vs. LPS). In contrast, treatment with matched platelet-poor plasma (PPP) at identical concentrations did not significantly modify cytokine release or gene expression patterns under inflammatory conditions. Together, these findings indicate that A2MG-EP counteracts inflammatory signalling and partially preserves the chondrocyte phenotype under both cytokine- and endotoxin-driven stimulation.

### 3.3. Effects in Equine Synoviocytes

**Figure 4:**
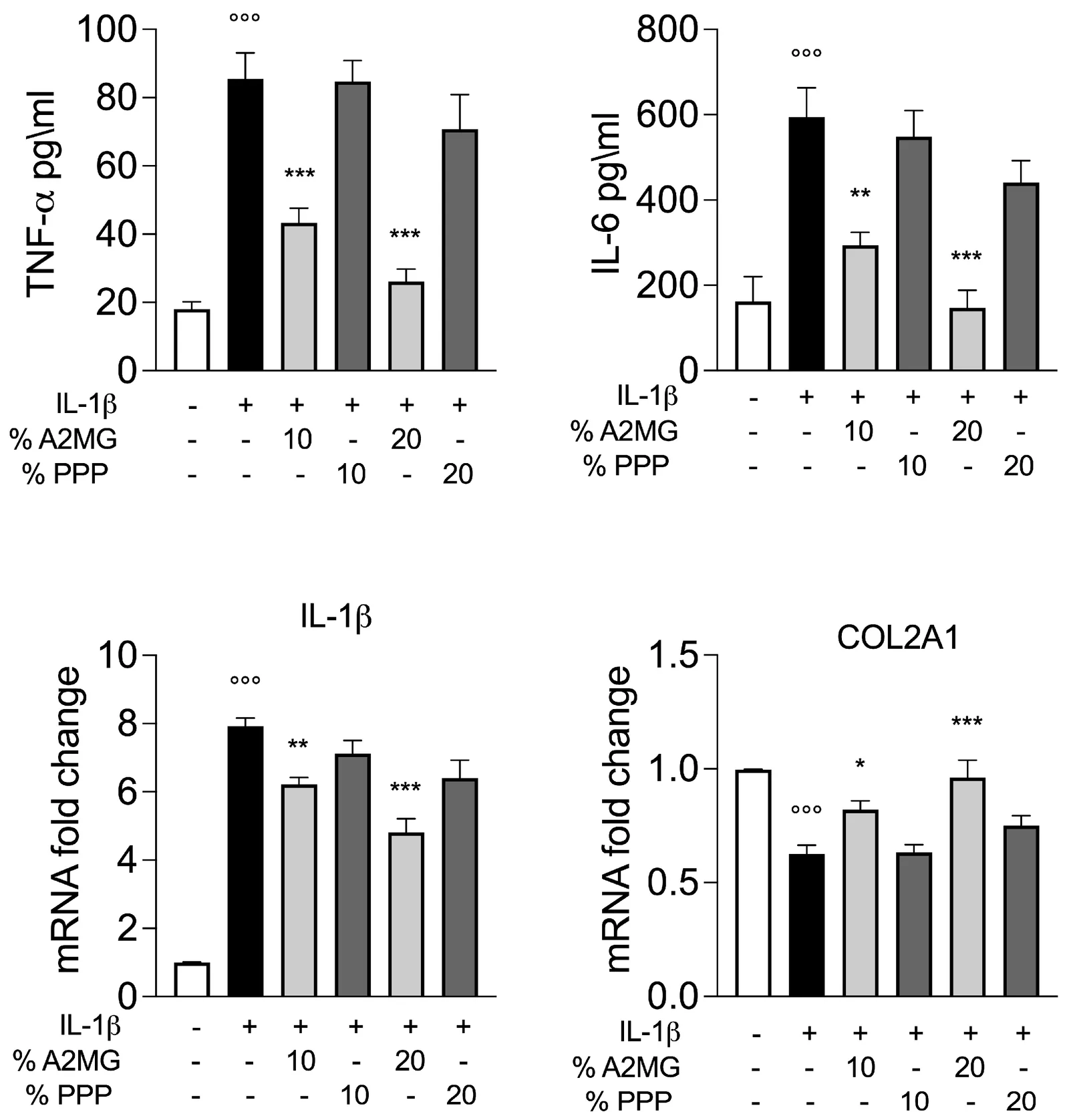
Effects of α_2_-macroglobulin-enriched plasma (A2MG-EP) on IL-1β-induced inflammatory responses in primary equine synoviocytes. Synoviocytes isolated from six independent donor horses (*n* = 6) were stimulated with IL-1β (2 ng/mL) for 24 h in the presence or absence of A2MG-EP or matched platelet-poor plasma (PPP) at 10% or 20% (*v*/*v*). TNF-α and IL-6 concentrations were measured in culture supernatants (upper panels). IL-1β and COL2A1 mRNA expression were determined by quantitative real-time polymerase chain reaction and expressed as fold change relative to untreated controls (lower panels). Statistical analysis was performed using a REML mixed-effects model with donor horse as the matching factor, followed by Geisser–Greenhouse correction and Tukey-adjusted multiple comparisons.°°° *p* < 0.001 vs. CTR; * *p* < 0.05 vs. IL-1β; ** *p* < 0.01 vs. IL-1β; *** *p* < 0.001 vs. IL-1β.

**Figure 5:**
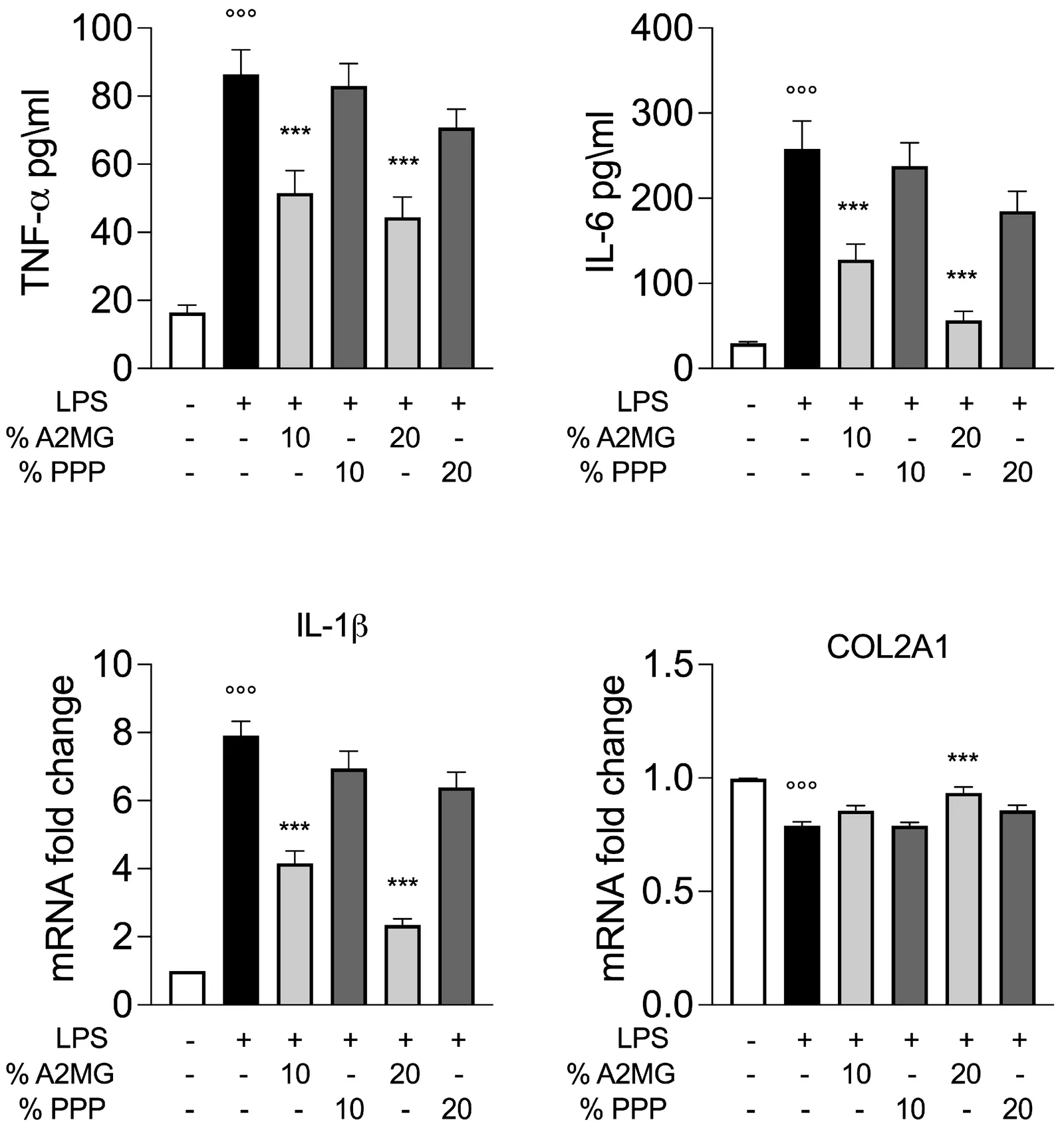
Effects of α_2_-macroglobulin-enriched plasma (A2MG-EP) on lipopolysaccharide-induced inflammatory responses in primary equine synoviocytes. Synoviocytes isolated from six independent donor horses (*n* = 6) were stimulated with lipopolysaccharide (10 ng/mL) for 24 h in the presence or absence of A2MG-EP or matched platelet-poor plasma (PPP) at 10% or 20% (*v*/*v*). TNF-α and IL-6 concentrations were measured in culture supernatants (upper panels). IL-1β and COL2A1 mRNA expression were determined by quantitative real-time polymerase chain reaction and expressed as fold change relative to untreated controls (lower panels). Data are presented as mean ± SEM. Statistical analysis was performed using a REML mixed-effects model with donor horse as the matching factor, followed by Geisser–Greenhouse correction and Tukey-adjusted multiple comparisons.°°° *p* < 0.001 vs. CTR; *** *p* < 0.001 vs. LPS.

Equine synoviocytes responded to both IL-1β and LPS stimulation with significant increases in TNF-α (*p* < 0.0001 vs. CTR) and IL-6 (*p* < 0.0001 vs. CTR) secretion together with elevated IL-1β mRNA expression (*p* < 0.0001 vs. CTR), confirming activation of a pro-inflammatory programme (Figure 4 and Figure 5). The magnitude of this response was lower than that observed in chondrocytes, consistent with the regulatory role of synoviocytes in joint inflammation. Co-treatment with α_2_-macroglobulin-enriched plasma (A2MG-EP) at 10% and 20% (*v*/*v*) significantly reduced cytokine secretion and suppressed IL-1β transcription in both inflammatory models. A2MG-EP 10% significantly reduced the secretion of TNF-α (*p* = 0.0009 vs. IL-1β; *p* = 0.0002 vs. LPS), IL-6 (*p* = 0.004 vs. IL-1β; *p* = 0.0007 vs. LPS) and IL-1β transcripts (*p* = 0.0061 vs. IL-1β; *p* < 0.0001 vs. LPS), compared with stimulated controls. The higher concentration of A2MG-EP (20% v/v) produced the strongest effect on TNF-α (*p* = 0.0004 vs. IL-1β; *p* < 0.0001 vs. LPS), IL-6 (*p* < 0.0001 vs. IL-1β; *p* = 0.0004 vs. LPS) and IL-1β expression (*p* < 0.0001 vs. IL-1β; *p* < 0.0001 vs. LPS). In contrast, matched platelet-poor plasma (PPP) did not significantly modify cytokine production or gene expression. Although COL2A1 expression was lower in synoviocytes than in chondrocytes, A2MG-EP increased COL2A1 expression under several experimental conditions. At 10%, A2MG-EP significantly increased COL2A1 expression in IL-1β-stimulated cells (*p* = 0.0402 vs. IL-1β), whereas no significant effect was observed in LPS-stimulated cells (*p* = 0.403 vs. LPS). At 20%, A2MG-EP significantly increased COL2A1 expression under both IL-1β- and LPS-stimulated conditions (*p* = 0.0009 vs. IL-1β, *p* = 0.007 vs. LPS). Collectively, these results indicate that the anti-inflammatory activity of A2MG-EP extends to synoviocytes, suggesting a broad modulatory effect on joint-associated cell types.

### 3.4. A2MG-EP Reduces MMP-13 Production in Inflamed Equine Joint Cells

**Figure 6:**
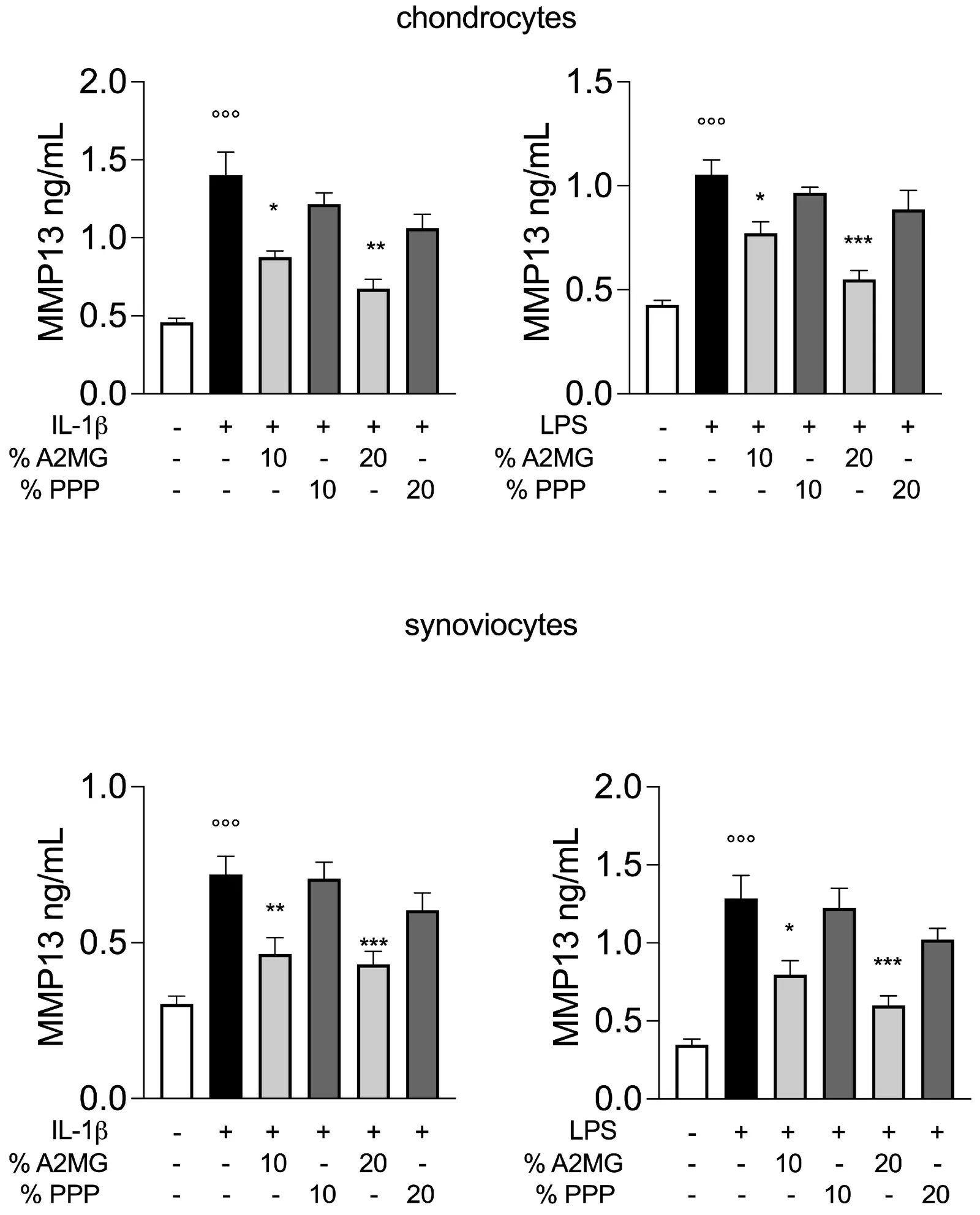
Effect of α_2_-macroglobulin-enriched plasma (A2MG-EP) on inflammation-induced MMP-13 production in primary equine chondrocytes and synoviocytes. MMP-13 concentrations were measured in culture supernatants from chondrocytes (upper panels) and synoviocytes (lower panels) isolated from six independent donor horses (*n* = 6) following stimulation with IL-1β (2 ng/mL) or lipopolysaccharide (LPS; 10 ng/mL) for 24 h in the presence or absence of A2MG-EP (10% or 20% *v*/*v*) or matched platelet-poor plasma (PPP; 10% or 20% *v*/*v*). Data are presented as mean ± SEM. Statistical analysis was performed using a REML mixed-effects model with donor horse as the matching factor, followed by Geisser–Greenhouse correction and Tukey-adjusted multiple comparisons.°°° *p* < 0.001 vs. control; * *p* < 0.05, ** *p* < 0.01 and *** *p* < 0.001 vs. stimulated cells.

Inflammatory stimulation significantly increased MMP-13 secretion in both primary equine chondrocytes and synoviocytes (Figure 6). Exposure of chondrocytes to IL-1β (2 ng/mL) for 24 h resulted in a marked elevation of MMP-13 levels compared with untreated controls (*p* < 0.001). A similar induction was observed following LPS stimulation (10 ng/mL) (*p* < 0.001), confirming activation of a catabolic response in these cells. Co-treatment with α_2_-macroglobulin-enriched plasma (A2MG-EP) significantly attenuated this response in a concentration-dependent manner. In chondrocytes, supplementation with 10% A2MG-EP produced a moderate reduction in MMP-13 release (*p* = 0.044 vs. IL-1β; *p* = 0.023 vs. LPS), whereas 20% A2MG-EP led to a more pronounced suppression of enzyme production (*p* = 0.005 vs. IL-1β; *p* = 0.0008 vs. LPS). In contrast, matched platelet-poor plasma (PPP) did not significantly modify MMP-13 levels under either stimulation condition. A comparable pattern was observed in synoviocytes, although the magnitude of the inflammatory response was lower than that detected in chondrocytes. Both IL-1β and LPS significantly increased MMP-13 secretion relative to controls, whereas A2MG-EP treatment reduced enzyme levels in a dose-dependent manner. At 10%, A2MG-EP significantly reduced MMP-13 secretion (*p* = 0.0014 vs. IL-1β; *p* = 0.015 vs. LPS), whereas the 20% concentration produced a greater inhibitory effect (*p* = 0.0008 vs. IL-1β; *p* = 0.0009 vs. LPS). Again, PPP failed to produce a measurable effect. Overall, these findings indicate that A2MG-EP effectively suppresses inflammation-driven production of the cartilage-degrading protease MMP-13 in equine joint cells.

### 3.5. A2MG-EP Mitigates Collagenase-Induced Matrix Degradation in Equine Tendon Explants

**Figure 7:**
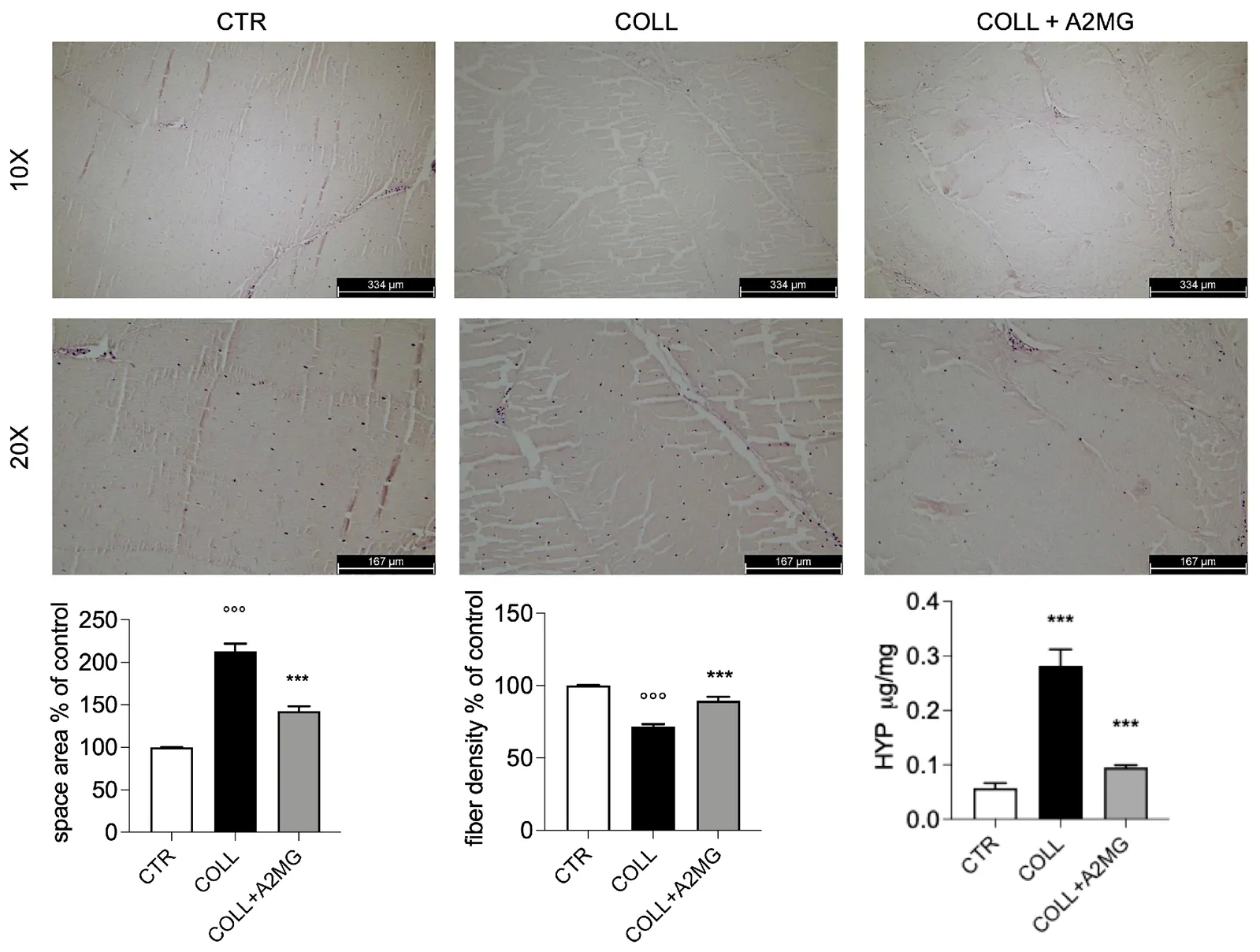
Effects of α_2_-macroglobulin-enriched plasma (A2MG-EP) on collagenase-induced extracellular matrix degradation in superficial digital flexor tendon explants obtained from six donor horses (*n* = 6). Representative haematoxylin and eosin-stained sections are shown together with quantitative histomorphometric and biochemical analyses. Black arrows indicate interfascicular spaces and areas of collagen fibre disruption. Collagenase-treated explants (COLL) exhibited fibre disorganization, matrix rarefaction, increased interfascicular space area and reduced fibre density compared with untreated controls (CTR). Co-treatment with A2MG-EP (COLL + A2MG) preserved tissue architecture and reduced histological damage. Quantitative graphs show interfascicular space area (%), normalized fibre density (% of untreated control) and hydroxyproline (HYP) release in culture supernatants normalized to tissue wet weight (μg/mg). Data are presented as mean ± SEM. Statistical analysis was performed using a REML mixed-effects model with donor horse as the matching factor, followed by Geisser–Greenhouse correction and Tukey-adjusted multiple comparisons.°°° *p* < 0.001 vs. CTR; *** *p* < 0.001 vs. COLL.

The tendon explant model was selected as a protease-dominant, collagen-rich system to functionally evaluate the anti-proteolytic activity of α_2_-macroglobulin at the tissue level, independently of joint-specific cellular responses. Histological examination of haematoxylin and eosin-stained sections revealed marked structural disruption following collagenase exposure. Compared with untreated controls, collagenase-treated explants exhibited pronounced fibre disorganization, widened interfascicular spaces and loss of overall matrix compactness (Figure 7), consistent with acute extracellular matrix degradation. Co-treatment with α_2_-macroglobulin-enriched plasma (A2MG-EP) substantially preserved tendon architecture. Explants exposed to collagenase in the presence of A2MG-EP showed a more parallel fibre organization, reduced interfascicular gaps and improved matrix homogeneity compared with the collagenase-only group. Quantitative image analysis confirmed these observations. Collagenase significantly increased interfascicular space area and reduced fibre density relative to control explants. Addition of A2MG-EP significantly attenuated these alterations, reducing interfascicular expansion and partially restoring fibre density toward baseline values. To biochemically corroborate the histological findings, collagen degradation was further assessed by measuring hydroxyproline release in culture supernatants normalized to tissue wet weight (Figure 7). Collagenase treatment induced a marked increase in hydroxyproline release compared with control explants, indicating extensive collagen breakdown. In contrast, co-treatment with A2MG-EP significantly reduced hydroxyproline levels, demonstrating a substantial inhibition of collagen degradation. Collectively, these results show that A2MG-EP protects tendon extracellular matrix integrity under acute proteolytic challenge, supporting a direct matrix-stabilizing and anti-proteolytic effect.

## 4. Discussion

Synovitis is a central driver of pain, effusion, and functional impairment in equine athletes, particularly in disciplines characterized by repetitive high-impact loading such as show jumping. Persistent inflammation of the synovial membrane promotes the sustained release of pro-inflammatory cytokines and matrix metalloproteinases (MMPs), accelerating cartilage degradation and contributing to the progression of degenerative joint disease (DJD) [26,27]. Although intra-articular corticosteroids and hyaluronic acid remain widely used in clinical practice, their effects are largely symptomatic and transient, and repeated corticosteroid administration has been associated with deleterious effects on cartilage integrity [28]. Consequently, there is increasing interest in biologic strategies capable of modulating upstream inflammatory and catabolic pathways rather than simply suppressing clinical signs. In this context, α_2_-macroglobulin (A2MG)—a large plasma glycoprotein with well-established anti-protease and cytokine-binding properties [1]—has emerged as a promising disease-modifying candidate. Acting as a broad-spectrum scavenger of both proteolytic enzymes and inflammatory mediators, A2MG has the potential to interrupt the self-perpetuating cycle of inflammation and matrix degradation that characterizes synovitis and early DJD [29,30,31,32,33]. While supportive evidence exists in human and small-animal models, data in the horse remain limited. The present study therefore sought to explore the biological effects of A2MG in experimental systems relevant to equine joint inflammation. Recent evidence further supports the emerging role of A2MG-based Orthobiologics in osteoarthritis management across species. A comprehensive narrative review [34] summarized the current mechanistic and translational evidence supporting A2MG as a biologic modulator capable of attenuating multiple inflammatory and catabolic pathways involved in OA progression. Experimental studies in rodent and porcine models demonstrated that intra-articular A2MG administration reduces synovial inflammation, decreases MMP-13, IL-1β, IL-6, and TNF-α expression, preserves cartilage structure, improves OARSI histopathology scores, and partially restores gait parameters. In vitro investigations have additionally shown that A2MG modulates NF-κB-dependent inflammatory signalling while promoting COL2A1 and aggrecan expression, supporting a broader chondroprotective effect beyond protease inhibition alone. These observations are consistent with our findings, in which A2MG attenuated inflammatory responses and preserved extracellular matrix organization in equine-derived experimental systems. Importantly, our study contributes preliminary equine-specific evidence supporting the biologic rationale of A2MG-enriched plasma in the context of synovitis and early degenerative joint disease. These findings are also consistent with our recent pilot clinical investigation in sport horses affected by chronic degenerative joint disease, in which intra-articular administration of an α_2_-macroglobulin-enriched plasma-derived orthobiologic preparation was associated with clinical improvement and favourable safety outcomes over the observation period [35]. Although clinical efficacy was not evaluated in the present experimental study, the convergence between the in vitro/ex vivo findings reported here and preliminary clinical observations supports the translational potential of A2MG-based orthobiologics for equine joint disease. Recent comparative analyses of equine blood-derived orthobiologics demonstrated substantial variability in A2MG concentration among commercially available systems, with differences likely influenced by device-specific processing methods as well as analytical quantification techniques, including ELISA- and mass spectrometry-based approaches [36]. Similar observations were recently reported by Van Reusel et al. [20], who demonstrated substantial compositional differences among standardized allogeneic equine orthobiologics, including A2MG-based products. Their study highlighted marked variability in protein content and biological composition among orthobiologic preparations, emphasizing the importance of product characterization and standardization when interpreting biological effects and comparing outcomes across studies, supporting the concept that different orthobiologic systems may exert distinct biological effects depending on their compositional characteristics. However, they also demonstrate that A2MG was not the only measurable difference between A2MG-EP and PPP, as total protein and residual platelet concentrations also differed. Accordingly, the present findings should be interpreted as biological effects of the complete A2MG-enriched plasma preparation rather than as direct evidence of the activity of purified A2MG. Across the in vitro models, A2MG-EP was well tolerated by primary equine chondrocytes and synoviocytes and consistently attenuated inflammatory responses induced by IL-1β and LPS. Treatment reduced the secretion of key inflammatory mediators, including TNF-α and IL-6, down-regulated IL-1β expression, and partially restored COL2A1 expression, a marker associated with chondrogenic stability and cartilage matrix maintenance [37,38,39]. These findings support the concept that A2MG contributes to cartilage homeostasis not only by neutralizing proteases, but also by limiting cytokine-driven inflammatory signalling [2]. Nevertheless, because A2MG-EP is a complex plasma-derived preparation, the present experiments do not establish that A2MG alone mediated these responses. Other processing-associated changes in plasma composition may have contributed to or interacted with A2MG in producing the observed effects. Notably, the magnitude of the response differed between cell types. Chondrocytes, which are directly responsible for maintaining cartilage extracellular matrix, exhibited a more pronounced protective response than synoviocytes. This likely reflects their greater exposure to catabolic stimuli and their dependence on tight regulation of protease and cytokine activity to preserve matrix integrity. Synoviocytes, while important amplifiers of inflammation, are less directly involved in structural matrix turnover, which may explain the comparatively modest response observed. This differential effect is consistent with previous reports demonstrating that A2MG binds IL-1β, TNF-α, and IL-6, thereby reducing their bioavailability and limiting NF-κB-dependent transcriptional activation [18,19]. The use of IL-1β and LPS provided complementary inflammatory challenges. IL-1β models cytokine-driven sterile inflammation through IL-1 receptor signalling and downstream NF-κB and MAPK activation, which are central to synovitis and cartilage catabolism. In contrast, LPS activates innate immune signalling primarily through the TLR4 pathway, producing a broader endotoxin-associated inflammatory response. The consistent attenuation of cytokine production, IL-1β expression, and MMP-13 release across both models indicates that the activity of A2MG-EP was not restricted to a single proximal inflammatory stimulus. These findings support a broader modulatory effect of the complete preparation but do not establish inhibition of any specific signalling pathway.

To complement the cellular findings with a tissue-level assessment, we employed an ex vivo tendon explant model. Tendon extracellular matrix is highly enriched in fibrillar collagen and particularly susceptible to protease-mediated degradation, making it a sensitive system in which to evaluate anti-proteolytic activity. In this model, collagenase exposure induced the expected features of acute matrix breakdown, including fiber disorganization and expansion of the interfascicular space. In contrast, co-treatment with A2MG-EP preserved collagen alignment and fibre density, providing tissue-level evidence of matrix-preserving activity within the complete A2MG-EP preparation [40,41]. Although this model was not intended to replicate joint pathology per se, it served as a reductionist, protease-dominant system to functionally validate the core biological action of A2MG at the tissue level. Beyond its mechanistic value, the tendon findings also suggest broader translational relevance, as protease-driven matrix degradation is a shared feature of both intra-articular soft tissues and tendon injuries in athletic horses. While tendon pathology was not the primary focus of the present study, these observations support further investigation of A2MG-based approaches in other musculoskeletal contexts. However, in the absence of experiments using purified A2MG, A2MG-depleted preparations, or selective neutralisation, the specific contribution of A2MG cannot be separated from that of other plasma constituents. At the same time, it is important to recognize the limitations inherent to simplified in vitro and ex vivo models. Although the present findings support the biological potential of A2MG-enriched plasma as an orthobiologic strategy for equine joint disease, several limitations should be acknowledged. First, the study was based on simplified in vitro and ex vivo experimental systems that cannot fully reproduce the complexity of the in vivo joint environment, including biomechanical loading, synovial fluid turnover, vascular interactions, and immune-cell recruitment. In addition, the absence of long-term in vivo follow-up and functional clinical outcome measures limits conclusions regarding the durability and clinical relevance of the observed anti-inflammatory and matrix-preserving effects.

Another limitation relates to the variability in A2MG quantification methods across studies, as differences in processing systems and analytical approaches, including ELISA- and mass spectrometry-based methods, may influence reported concentrations and complicate direct comparison among orthobiologic products. Moreover, biological variability among donor horses, together with potential variability introduced during preparation and processing of A2MG-enriched plasma, may influence the final composition and biological activity of the product. Factors such as individual inflammatory status, physiological condition, blood composition, and centrifugation or incubation protocols could contribute to inter-sample variability. Nevertheless, the present study provides preliminary equine-specific evidence supporting the rationale for further investigation of A2MG-based therapies in synovitis and early degenerative joint disease.

## 5. Conclusions

This study provides preliminary evidence that a characterised, pooled allogeneic A2MG-enriched plasma preparation has anti-inflammatory and matrix-preserving activity in complementary equine musculoskeletal models. A2MG-EP attenuated inflammatory responses in primary equine chondrocytes and synoviocytes and reduced collagenase-mediated extracellular matrix degradation in tendon explants. A2MG-EP is a complex plasma-derived product, and its characterisation did not encompass other potentially bioactive constituents. The observed effects therefore cannot be attributed exclusively to A2MG and should be interpreted as the biological activity of the complete A2MG-enriched plasma preparation. These findings align with a broader therapeutic perspective that seeks to enhance endogenous pro-homeostatic pathways rather than merely suppressing symptoms. From this standpoint, A2MG may be viewed as a prototype regulatory molecule capable of rebalancing the joint microenvironment while preserving physiological repair processes. Future studies should focus on controlled, randomized clinical trials with extended follow-up to determine whether the protective effects observed here translate into durable clinical benefit.

## Figures and Tables

**Table 1:** SsoAdvanced Universal SYBR Green Supermix.

Gene	Forward (5′–3′)	Reverse (5′–3′)
IL-1β	GGCCTCAAGGAAAAGAACCT	TGGGGTACATTGCAGACTCA
COL2A1	GCCCGTCTGCTTCTTGTAATA	CGTGACTGGGATTGGAAAGT
GAPDH	GGCAAGTTCCATGGCACAGT	CACAACATATTCAGCACCAGCAT

## Data Availability

The original contributions presented in this study are included in the article. Further inquiries can be directed to the corresponding author.
